# Anti-doping literacy scale (ADL-S): development and validation of a French language instrument based on the doping awareness literacy framework

**DOI:** 10.3389/fspor.2026.1810415

**Published:** 2026-09-15

**Authors:** Thomas Lanteri, Fabienne d’Arripe-Longueville, Camille Paris, Éric Meinadier, Karine Corrion

**Affiliations:** 1 LAMHESS, Université Côte d'Azur, Nice, France; 2 Medical Department, French Federation of Cycling, Paris, France

**Keywords:** anti-doping literacy, athlete education, prevention, psychometric validation, scale development

## Abstract

Doping prevention has increasingly shifted toward education as a central strategy for promoting clean sport. This evolution reflects a growing consensus that effective prevention must go beyond the transmission of rules and sanctions and instead develop athletes’ competencies to make informed, autonomous, and ethically grounded decisions. Doping Awareness Literacy (DAL) framework defines anti-doping literacy as comprising three complementary dimensions: (1) functional literacy (i.e., knowledge of anti-doping rules and associated health risks); (2) interactive literacy (i.e., ability to use this information in training and social contexts); and (3) critical literacy (i.e., capacity to evaluate information and resist doping-related pressures). Despite its theoretical relevance, validated instruments assessing specifically these dimensions remain scarce, particularly in non-English-speaking contexts. The study aimed to develop and validate the Anti-Doping Literacy Scale (ADL-S), a French-language instrument based on the DAL framework, designed to provide both general anti-doping literacy and domain-specific literacy competencies. 265 adult athletes participated in a four-phase validation process: (1) Phase 1 involved item generation based on the DAL model and international anti-doping education guidelines, followed by expert review to ensure content validity and conceptual clarity; (2) Phase 2 examined the factorial structure and internal consistency using confirmatory factor analyses; (3) Phase 3 explored ADL-S external correlates through associations with psychosocial constructs relevant to doping, including moral disengagement and resistive self-regulatory efficacy; (4) Phase 4 evaluated test–retest reliability over time. Final version of ADL-S comprises 21 items distributed across three subscales reflecting functional, interactive, and critical anti-doping literacy. Confirmatory factor analyses supported a bifactor structure, indicating the coexistence of a general anti-doping literacy factor and specific subdimensions, with satisfactory model fit indices and internal reliability coefficients. Exploratory analyses showed negative associations with moral disengagement and positive associations with resistive self-regulatory efficacy, consistent with theoretical expectations. Test–retest analyses demonstrated satisfactory temporal stability. Overall, ADL-S shows robust psychometric properties and provides a theoretically grounded tool for assessing anti-doping literacy in French-speaking athletic populations. Beyond measurement, this instrument offers practical value for evaluating and guiding education-based prevention programmes aligned with international anti-doping standards, supporting the development of informed, autonomous, and health-protective decision-making among athletes.

## Introduction

Doping prevention today increasingly relies on education as a cornerstone for promoting clean sport, as formalised by the International Standard for Education (ISE) developed by World Anti-Doping Agency (WADA) in 2021 (1). This shift reflects a growing consensus, supported by both WADA and UNESCO, that athletes need more than rules and sanctions to guide their behaviour (2). They require educational approaches that foster personal values, ethical reasoning, and the capacity to make informed and autonomous decisions (1, 3, 4). The ISE marks a significant step in this evolution by establishing educational objectives that go beyond mere rule comprehension, aiming to equip athletes with the competence and integrity needed to navigate the complex challenges of high-performance sport. This educational shift is particularly relevant given the multifactorial nature of doping behaviour (5). Athletes’ decisions to engage in doping may reflect coping mechanisms in response to injury, performance pressure or psychological vulnerability (6). Rather than being solely attributable to individual factors, anti-doping rule violations often emerge from complex interactions between athletes and complex competitive contexts (6–8). In this vein, Filleul et al. have shown that doping behaviours among elite cyclists may emerge from situations of vulnerability, such as physical exhaustion, loss of confidence or relational tensions, illustrating how doping may be perceived as a form of adaptation rather than transgression (9). In light of this complexity, educational approaches must go beyond the transmission of rules and sanctions to empower athletes with the knowledge and skills needed to navigate risk situations. Anti-doping education aims not only to improve rule comprehension, but also to support informed decision-making, risk appraisal, ethical reflection, resistance to social pressures, and protective behaviours toward oneself and others. Providing comprehensive information on anti-doping regulations is essential (6), but not sufficient.

The concept of literacy, defined as an individual's ability to access, understand, and apply information to manage risk situations (10), offers a broader framework for developing athletes’ competencies. Literacy refers to a progressive set of capacities enabling individuals to access, understand, communicate, appraise, and use information to support informed action (10). In this sense, literacy shifts the focus from information transmission alone toward the development of competencies that support autonomous and responsible decision-making. Applied to anti-doping, this perspective moves beyond the delivery of rules, prohibited substances, and testing procedures, and instead emphasises athletes’ ability to understand anti-doping information, discuss uncertainties, make informed decisions, and critically engage with the social and performance-related pressures that may shape doping-related situations (11). This conceptual transfer was formalised through the SAFE YOU and SAFE YOU + programmes (11) which introduced Doping Awareness Literacy (DAL) as a competency-based educational framework. SAFE YOU programme conceptualises athlete development as a progression from understanding information (Knowing), to applying this information in decision-making situations (Deciding), and ultimately to critically engaging with and influencing the sporting environment (Leading) (11). This progression reflects the assumption that effective anti-doping education requires more than factual knowledge alone. Athletes must not only understand anti-doping information but also be able to apply it in everyday decisions and, ultimately, critically engage with and positively influence their sporting environment. Accordingly, in line with Nutbeam's model, the Knowing stage corresponds primarily to functional literacy, the Deciding stage to interactive literacy, and the Leading stage to critical literacy. These levels reflect the development of athletes’ skills to understand, engage with and make autonomous decisions about anti-doping issues (12).

Anti-doping literacy does not develop in isolation but within a broader performance context that may shape athletes’ opportunities to access, use, and critically evaluate information relevant to clean sport (6). This perspective is consistent with health literacy frameworks, which conceptualise literacy not only as an individual competency but also as a product of interactions between individuals and their informational and social environments (10, 13). At the functional literacy level, athletes’ understanding of anti-doping information may be influenced by the accessibility, quality, consistency, and credibility of available information sources. Exposure to contradictory messages, misinformation, or difficulties identifying trustworthy sources may complicate the acquisition of accurate knowledge (10, 13). At the interactive literacy level, athletes may differ in their opportunities to discuss anti-doping issues, seek clarification, and apply information in concrete situations. Supportive interactions with coaches, athlete support personnel, and other members of the athlete entourage may facilitate the practical mobilisation of anti-doping knowledge and the development of informed decision-making skills (11). At the critical literacy level, broader organisational and cultural factors may become particularly relevant. Belief in anti-doping institutions, perceptions of legitimacy and fairness, opportunities to raise concerns, and the ability to critically evaluate information sources may all influence athletes’ capacity to make independent judgements regarding anti-doping-related situations (11).

In this context, DAL provides an evaluative framework for examining whether and to what extent, anti-doping education moves beyond knowledge transmission toward the development of applied and critical literacy capacities. Historically, anti-doping education has focused primarily on information provision, awareness raising, anti-doping rules, prohibited substances, health risks, testing procedures, sanctions, and athletes’ responsibilities (1, 3). Although more recent initiatives, including the SAFE YOU and SAFE YOU + programmes, have promoted broader educational objectives related to decision-making, autonomy, and personal responsibility (11), the systematic development and assessment of interactive and critical literacy capacities remain comparatively limited. This framing positions DAL as a construct concerned not only with understanding anti-doping information, but also with using, discussing, and critically evaluating it across sporting contexts.

However, as emphasised by several researchers (14, 15), evaluating the actual impact of such educational programmes remains challenging. Most existing measurement tools focus on attitudes, beliefs or intentions (15), but few are designed to assess literacy levels across the functional, interactive and critical dimensions described in the DAL framework. The GRADE IT questionnaire, developed by Blank et al. (12) represents an important DAL-based impact-evaluation instrument for anti-doping education, operationalised through knowledge items and scale-based judgements. Comprising 52 items, it assesses the three DAL stages (i.e., functional, interactive, and critical literacy) alongside other relevant dimensions such as perceived trust and perceived legitimacy. GRADE IT provides a comprehensive and nuanced assessment of athletes’ educational development in relation to anti-doping, moving beyond simple knowledge testing to measure their readiness to act responsibly in applied situations. Although GRADE IT incorporates the DAL stages, it was primarily developed as a broader educational impact-evaluation framework that also encompasses dimensions such as trust and legitimacy. To date, no instrument has been specifically developed to assess anti-doping literacy exclusively through its functional, interactive, and critical dimensions. A narrower focus should examine whether the different dimensions of literacy can be represented as components of a common latent concept and provide both a general indicator of anti-doping literacy and domain-specific literacy scores.

Therefore, the present study aimed to address this gap by developing and validating, ADL-S, a French-language instrument providing a complementary psychometric operationalisation of the Doping Awareness Literacy (DAL) framework. The objective of the present study was not to adapt GRADE IT into French, but to develop a complementary instrument specifically focused on the psychometric assessment of anti-doping literacy itself. Although DAL provides a conceptual framework describing how athletes progress from acquiring anti-doping knowledge, to applying it in decision-making and ultimately fostering a clean sport environment (16), few studies have investigated whether these three levels can be conceptualised and measured as a unified psychometric construct. Accordingly, the ADL-S aims to operationalise functional, interactive and critical literacy within a dedicated psychometric instrument, designed to provide both general anti-doping literacy and domain-specific literacy competencies. The scale may also be a useful resource for identifying educational needs, guiding targeted interventions, and supporting the monitoring and evaluation of anti-doping education programmes.

## Phase 1—Preliminary version

The aim of phase 1 was to develop a preliminary French version of the ADL-S questionnaire. The item generation process was guided by the French version of the GRADE IT tool and by literature on vulnerable situations that may predispose athletes to consider doping (9).

### Methods

The item construction process was based on the conceptual framework of the DAL model (12), which defines literacy across three progressive levels, or domains. Functional literacy focused on athletes’ basic knowledge of anti-doping rules and consequences. Interactive literacy dealt with their capacity to handle situations where doping might be a risk (9, 12). Critical literacy referred to their confidence to assess doping-related information and make independent, ethical decisions. To ensure consistency and clarity, each item was formulated with a specific introductory phrase that corresponded to its target literacy level: “For each statement, rate how well it reflects your knowledge/capacity/confidence, from 1 (not at all) to 6 (completely)”. Items addressing functional literacy began with “I understand,” “I know,” or “I am aware.” Items focusing on interactive literacy began with “I feel capable of,” while those related to critical literacy began with “I am confident”.

The questionnaire was then constructed following a two-step expert review procedure, as recommended for developing scales (17, 18). In the first step, a panel of five experts (three researchers in sport psychology, one PhD student and one sports medicine doctor) reviewed and refined the relevance of the questionnaire items. In the second step, a broader panel of sixteen experts (ten PhD students, four researchers and two anti-doping educators) evaluated the clarity of each item using a 6-point Likert scale (1 = not at all clear, 6 = completely clear), in accordance with the guidelines proposed by Simms et al. (19). Experts were also invited to provide qualitative feedback and suggestions for improvement.

The mean clarity score was calculated for each item and literacy domain from the expert ratings. A minimum mean score of 5 was then used as the threshold for item clarity, in line with common practice in tool development using Likert-type scales (19, 20).

### Results and discussion

The initial version of the questionnaire consisted of 32 items. Following the review by the panel of five experts, 23 items were retained based on their relevance and clarity, ensuring the overall quality of the tool (17) and close alignment with the DAL theoretical model (12). The clarity of the 23 items was considered satisfactory as all items scored greater than 5 out of 6 when averaged over the expert panel, and the overall mean clarity was 5.51/6 (*SD* = .32). The final version of the preliminary ADL-S questionnaire thus includes 23 items, grouped into three subscales: 8 items for functional literacy, 10 for interactive literacy, and 5 for critical literacy (see Supplementary Table in Appendix).

## Phase 2 - Factorial structure and reliability

The aim of phase 2 was to examine the factorial structure and reliability of the ADL-S questionnaire (17, 18).

### Methods

#### Participants

A total of 1,329 participants initiated the survey. Of these, 315 fully completed the survey and met the inclusion criteria (i.e., over 16 years of age, fluent in French, and self-reporting current or former involvement in competitive sport). The eligible sample had a mean age of 30 years, included 61% male and 38% female participants, and was predominantly composed of athletes reporting more than 6 h of training per week. After data quality screening, including instructed-response attention-check items in which participants were asked to select a specific response option, for example “Tick the box corresponding to ‘a little’” (2), 265 self-reporting current or former competitive athletes were retained for the final analyses. This final analytic sample had a mean age of 28 years, included 66% male and 34% female participants, and also predominantly comprised athletes reporting more than 6 h of training per week. Recruitment was carried out via three sources: (a) the Prolific platform (*n* = 207; *M*_age_ = 30 years; 133 males and 74 females), (b) a sports science university (*n* = 46; *M*_age_ = 21 years; 29 males and 17 females), and (c) a rugby club training centre (*n* = 12; *M*_age_ = 23 years; 12 males). The final sample included 174 males and 91 females (*M*_age_ = 28 years; *SD* = 10) representing a wide variety of sports, including both individual (*n* = 112) and team disciplines (*n* = 153). This sample size is consistent with established recommendations for confirmatory factor analysis (17, 18).

#### Procedure

Data were collected online using the LimeSurvey platform hosted by University. Before beginning the questionnaire, a pre-screening survey ensured that only eligible participants were included; those who did not meet the inclusion criteria were excluded. Each participant was assigned an anonymous identification code, and no missing data were permitted (21). The average completion time for the ADL-S questionnaire was approximately 10 min.

To assess the factorial structure of the questionnaire, a series of Confirmatory Factor Analyses (CFA) were conducted, based on the three distinct dimensions of literacy in the DAL model (i.e., functional, interactive and critical). Following the procedure outlined by Myers et al. (22), four competing models were tested: (a) a unidimensional model in which all items loaded onto a single literacy factor (model a); (b) a first-order three-factor model reflecting the three levels of literacy (model b); (c) a second-order hierarchical model in which the three first-order factors loaded onto a higher-order global literacy factor (model c); and (d) a bifactor model specifying one general factor alongside the three specific factors (model d).

The model fit was evaluated using indices commonly used in the scientific literature (17, 18). The minimum chi-square test was calculated (*χ*^2^/*df*), with values below 2 indicating a good fit between the hypothesized model and the observed data (23). In line with established guidelines (17, 18), the following indices were also considered: (a) the Root Mean Square Error of Approximation (RMSEA), with values <.05 and a 90% confidence interval upper bound <.08; (b) the Tucker–Lewis Index (TLI); and (c) the Comparative Fit Index (CFI), with values >.90 considered acceptable for the latter two indices. To support scoring interpretation (i.e., general and specific factors), specific indices were calculated based on the retained model (i.e., *ωh*, ECV and PUC; 24).

The reliability of the ADL-S questionnaire was evaluated using McDonald's omega (*ω*), which is considered a more accurate measure of internal consistency than Cronbach's alpha, particularly for multidimensional scales (24). Omega coefficients were computed for each of the three literacy dimensions (i.e., functional, interactive, and critical) with values equal to or higher than .70 considered satisfactory.

### Results and discussion

Model fit indices for the series of factorial analyses are presented in Table 1. In the initial CFA, the bifactor model demonstrated the most satisfactory fit indices, *χ*^2^(201) = 320.16; *p* < .001; TLI = .94; CFI = .95; RMSEA = .04; RMSEA LO/HI = .03/.05. However, two negatively worded items with reversed response scaling exhibited negative factor loadings. Examination of the response patterns suggested that these loadings may have resulted from participant misinterpretation of the reverse-scored format rather than from conceptual inconsistency (see in Supplementary Table in Appendix). A revised CFA was then conducted on the resulting 21-item version of the questionnaire. Once again, the confirmatory bifactor model demonstrated the best fit indices: *χ*^2^(162) = 200.94; *p* = .02; TLI = .98; CFI = .98; RMSEA = .03; RMSEA LO/HI = .01/.04, confirming the robustness of this factorial structure. All items, along with their standardized factor loadings by subscale, are presented in Figure 1. Modification indices suggested residual covariances between items with similar wording and closely related content (25). One residual covariance involved two functional literacy items, LF3 and LF4, which both refer to understanding key anti-doping administrative procedures: Therapeutic Use Exemptions (TUEs) and the Anti-Doping Administration and Management System (ADAMS). Two additional residual covariances were identified within the interactive literacy domain: LI6 with LI8, and LI7 with LI8. These items share a common focus on athletes’ perceived ability to resist contextual pressures or performance-related difficulties without being tempted to use prohibited substances and/or methods.

**Table 1:** Confirmatory factor analysis (CFA): summary of fit indices for the ADL-S scale.

Competing models	*χ^2^* (*df*)^a^	*p* ^b^	RMSEA^c^ [90% CI]	TLI^d^	CFI^e^
Model a	1,065.19 (186)	<.001	.134 [.123–.142]	.600	.646
Model b	286.24 (183)	<.001	.046 [.036–.056]	.952	.958
Model c	286.24 (183)	<.001	.046 [.036–.056]	.952	.958
Model d	200.943 (162)	<.020	.030 [.013–.046]	.980	.984

a Chi-square and degree of freedom.

b *p*-value.

c Root Mean Square Error of Approximation.

d Tucker–Lewis index.

e Comparative Fit Index.

Model a: unidimensional model; Model b: first-order model with three correlated factors; Model c: second-order hierarchical model; Model d: confirmatory bifactor model.

**Figure 1:**
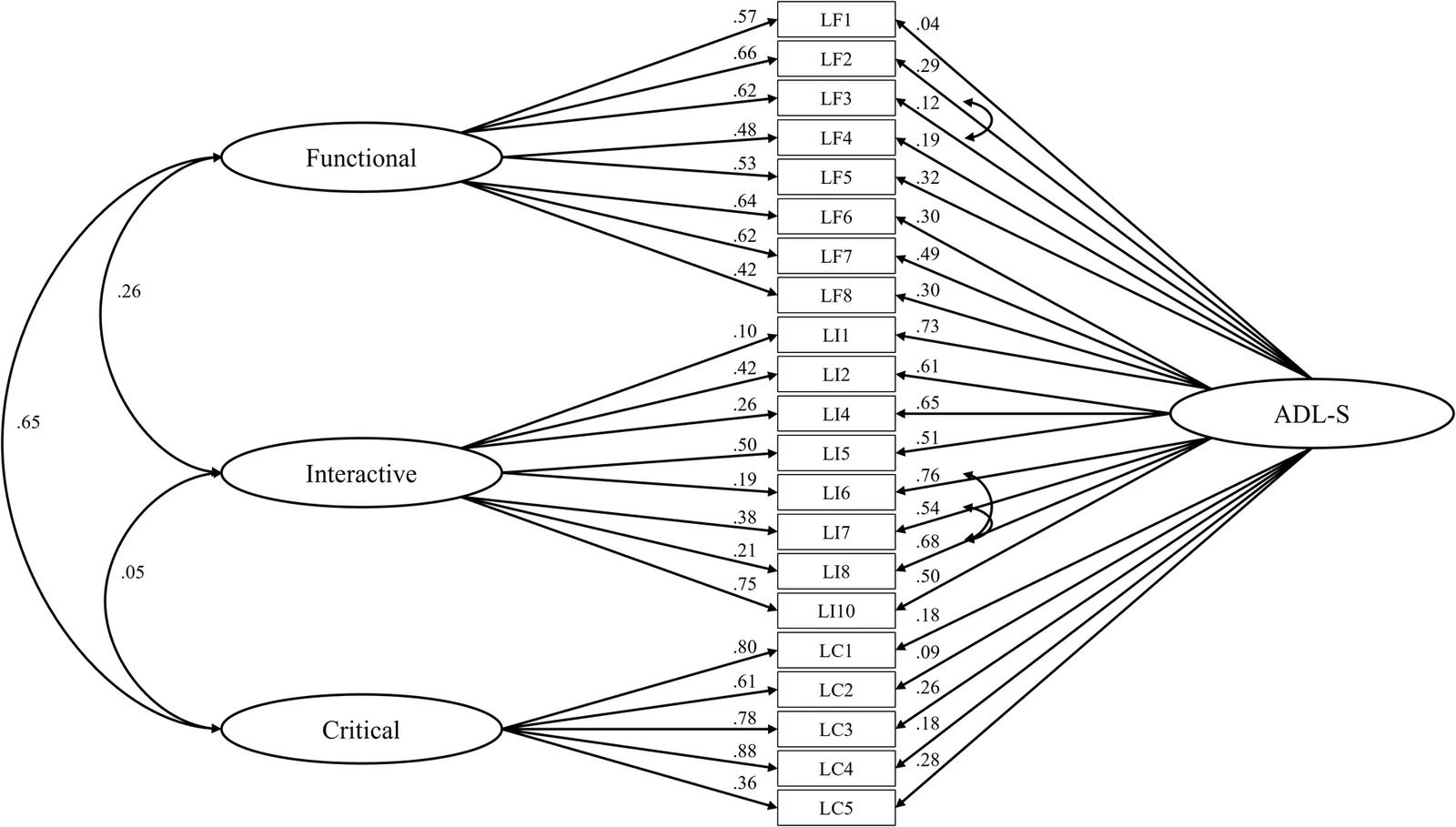
Bifactor confirmatory model of the ADL-S scale.

The computation of McDonald's omega demonstrated good reliability across all subscales, with *ω* = .81 for functional literacy, *ω* = .89 for interactive literacy, *ω* = .84 for critical literacy, and *ω* = .91 for the general factor. These values exceed the commonly used threshold of .70, suggesting strong internal consistency for both the specific dimensions (24) and the overall construct measured by the ADL-S questionnaire. Specific bifactor indices were examined to guide the interpretation of the retained bifactor model. Omega hierarchical indicated that a substantial proportion of reliable variance was attributable to the general factor (*ωH* = .662). The explained common variance (ECV = .481) and percentage of uncontaminated correlations (PUC = .686) suggested that the general factor was meaningful, while the specific dimensions retained interpretative value.

Taken together, these results provide robust psychometric support for the structural reliability of the ADL-S. The bifactor model provides a useful framework for examining whether anti-doping literacy can be represented by both a general literacy factor and domain-specific components (26, 27). Conceptually, the general factor was interpreted as general anti-doping literacy, reflecting athletes’ overall capacity to understand, use, and critically appraise anti-doping information across sport-related situations. This general factor does not erase the distinction between functional, interactive, and critical literacy, but captures the common literacy-related competence shared by these three levels. These subscale scores provide complementary information on specific literacy profiles, particularly for identifying educational needs and guiding targeted interventions.

## Phase 3 - Exploratory external correlates

This phase aimed to explore the external correlates of the ADL-S by examining its associations with broader psychosocial constructs relevant to doping-related decision-making. Specifically, moral disengagement and resistive self-regulatory efficacy were selected because previous research has identified them as socio-cognitive processes associated with doping-related outcomes, including doping susceptibility and likelihood (28, 29). The objective was to examine how the ADL-S fits within a broader psychosocial context of anti-doping by exploring its relationships with these constructs.

Anti-doping literacy extends beyond factual knowledge to encompass competencies involved in interpreting and applying anti-doping information in decision-making contexts (11). In this perspective, exploring its relationships with constructs reflecting how athletes regulate their behaviour when confronted with morally questionable conduct or pressures to dope may contribute to a broader understanding of the positioning of anti-doping literacy in such situations. Moral disengagement refers to cognitive mechanisms through which individuals may justify behaviours that conflict with moral standards (30, 31), whereas resistive self-regulatory efficacy refers to athletes’ perceived capacity to resist doping-related pressures (32, 33). These constructs were included because they represent distinct psychosocial processes relevant to transgressive sport contexts which may include doping-risk situations. However, neither construct directly assesses literacy, knowledge, or competence, and the available literature does not establish a necessary relationship between them and anti-doping literacy. Their associations with ADL-S scores were therefore examined on an exploratory basis.

### Methods

The same 265 participants from the previous phase completed two additional validated self-report questionnaires in French.

#### Measures

To assess moral disengagement, we used the Moral Disengagement in Sport Short Scale (ECDMS; 31). This six-item tool comprises two subscales: *projection of fault onto others or sharing of responsibility* (ECDMS_RPR), and *minimization of transgression and their consequences* (ECDMS_MIN). Responses are rated on a 7-point Likert scale ranging from 1 (“not at all agree”) to 7 (“totally agree”). Previous research has demonstrated acceptable model fit and internal consistency of both subscales, supporting the psychometric properties of the ECDMS in sport contexts (33, 34).

The Resistive Self-Regulatory Efficacy in Sport Scale (*RSRESS*; 35); is a six-item scale designed to assess athletes’ perceived ability to resist social pressure to engage in unethical behaviours such as cheating, aggression, or doping. The scale comprises a single dimension and uses a 7-point Likert response format from 1 (“not at all capable”) to 7 (“completely capable”). Previous research has reported a satisfactory factorial structure and strong internal consistency, supporting the reliability and construct validity of the RSRESS in sport-related contexts (33, 35).

These instruments were selected because validated French-language versions with established psychometric properties were available and because they assessed moral disengagement and resistive self-regulatory efficacy within the broader sport context. This allowed us to examine moral and self-regulatory processes relevant to athletes’ responses to risk, pressure, and transgressive situations in sport.

#### Statistical analysis

The external correlates of the ADL-S were assessed using bivariate Pearson correlations between the ADL-S subscales (i.e., functional, interactive, and critical literacy) and the two theoretically related constructs measured by the ECDMS subscales and the RSRESS score. The strength of the correlations was interpreted following the guidelines proposed by Schober et al. (36): .00–.10 = negligible; .10–.39 = weak; .40–.69 = moderate; .70–.89 = strong; .90–1.00 = very strong.

### Results and discussion

Our results revealed a general negative correlation between anti-doping literacy and moral disengagement, as well as a positive correlation between anti-doping literacy and resistive self-regulatory efficacy. At the subscale level, interactive literacy showed the strongest negative correlations with moral disengagement (*r* = −.309 with ECDMS_MIN; *r* = −.226 with ECDMS_RPR, both *p* < .001), followed by critical literacy (*r* = −.134, *p* < .01 and −.217, *p* < .05). Functional literacy was not significantly associated with either dimension. Conversely, all subscales were positively correlated with resistive self-regulatory efficacy, with interactive literacy again showing the strongest association (*r* = .477, *p* < .01), followed by functional literacy (*r* = .329, *p* < .01) and critical literacy (*r* = .293, *p* < .01). The global ADL-S score was significantly associated with the two theoretically expected constructs, displaying negative correlations with moral disengagement (*r* = −.235 and −.239), and a positive correlation with resistive self-regulatory efficacy (*r* = .502), all *p* < .01. The observed correlations provide preliminary insights into how ADL-S scores relate to broader psychosocial processes involved in doping-risk situations. Rather than constituting direct evidence of construct validity, these associations contribute to an initial exploration of the broader nomological context surrounding anti-doping literacy. Specifically, athletes reporting higher anti-doping literacy also tended to report lower levels of moral disengagement and higher levels of resistive self-regulatory efficacy. However, the nature and direction of these relationships remain to be established.

## Phase 4 - Temporal stability

The aim of this phase was to assess the test-retest reliability of the questionnaire, by checking whether the instrument produces consistent results over time. For self-report tools, it is important that scores stay stable when the same people complete the questionnaire again, showing that any changes are due to real differences and not random or time-related factors (17, 18, 37).

### Methods

A subsample of 67 current or former competitive athletes (44 males and 23 females, *M*_age_ = 31 years, *SD* *=* 10 years) completed the questionnaire a second time after approximately 14 days, an interval chosen to minimise recall bias while limiting the likelihood of true changes in literacy levels (38). With 67 participants for 21 items (ratio 1:3), this subsample met the recommended item-to-participant ratio (1:1–1:4) for test–retest reliability studies (38). Temporal stability was first assessed by comparing the mean scores of each subscale (i.e., functional, interactive, and critical literacy) between Time 1 and Time 2 using paired-sample *t*-tests. A second comparison was made by calculating intraclass correlation coefficients (ICCs) for the global score and for each subscale (i.e., functional, interactive, and critical literacy).

### Results and discussion

The paired-sample *t*-tests showed no significant differences between the two time points for any of the subscales or the total score. However, ICC analyses indicated low reliability. To examine whether extreme values influenced the results, individual change scores (Δ = T2 − T1) were computed and inspected using boxplots, an approach recommended to detect unusual intra-individual variability (39). Following Tukey's rule, values below Q1 − 1.5 × IQR or above Q3 + 1.5 × IQR were considered outliers (40, 41). Six participants (three males and three females) with extreme individual change scores were excluded. The new analyses performed on the reduced sample (*n* = 61) provided better *t-*tests results and good ICC values, reaching satisfactory levels of temporal reliability. Detailed mean score comparisons and ICC values are presented in Table 2. These findings indicate that the ADL-S demonstrates satisfactory temporal stability over a two-week interval, supporting its test-retest reliability. This reinforces its suitability for longitudinal or intervention studies, where measurement consistency is essential to ensure that observed changes reflect real changes in knowledge or attitudes (17, 18).

**Table 2:** Estimates of the temporal stability.

Global score and subscale	Time 1	Time 2	ICC [95% CI]^c^	t^d^ (df)^e^
	M^a^ (*SD*^b^)	M (*SD*)		
ADL-S (*n* = 67)	5.018 (.585)	4.918 (.672)	.484^◊^ [.278–.648]	1.277^▴^ (66)
ADL-S (*n* *=* *61*)	4.992 (.591)	4.998 (.568)	.772^◊^ [.647–.857]	−.117* (60)
Functional	4.973 (.708)	4.988 (.679)	.697^◊^ [.541–.807]	−.203* (60)
Interactive	5.373 (.594)	5.350 (.624)	.592^◊^ [.400–.743]	.322* (60)
Critical	4.420 (.922)	4.459 (.861)	.662^◊^ [.493–.782]	−.417* (60)

a Mean.

b Standard deviation.

c Intraclass correlation coefficients.

d Student's *t*-test statistic.

e Degree of freedom.

▴ *p* < .05.

* *p* > .60.

◊ *p* < .001.

## Discussion

This study aimed to develop and validate the ADL-S questionnaire, a French-language tool designed to assess anti-doping literacy, based on the Doping Awareness Literacy (DAL) framework (12). The final version comprises 21 items across three dimensions: functional, interactive, and critical literacy (12, 13).

ADL-S adhered to rigorous methodological standards (17, 18), and showed item clarity, relevance, and theoretical coherence. Confirmatory factor analysis supported a bifactor model capturing both a global literacy score and three distinct (i.e., functional, interactive, critical) but interconnected subscales (27). Moreover, ADL-S demonstrated satisfactory temporal stability, providing evidence of its reliability and supporting its suitability for use in longitudinal designs (17, 18).

ADL-S addresses a methodological gap in the specific measurement of anti-doping literacy by offering a validated multidimensional psychometric tool to assess knowledge and skills in anti-doping contexts. While GRADE IT represented an initial attempt to operationalise anti-doping literacy, it did not focus solely on literacy, its validation remained partial, and its primary function was educational (12). ADL-S builds on GRADE IT by also incorporating realistic doping-related items (9) and complements it by following established scale development protocols (17, 18). The bifactor model and the associated bifactor indices support a two-level interpretation of ADL-S scores. The total score showed high reliability (i.e., *ω*), and the general factor accounted for a substantial proportion of reliable variance (i.e., *ωH*), enabling the use of a global anti-doping literacy score (26, 27). This score provides an overall picture of athletes’ capacity to understand, use, and critically appraise anti-doping information across sport-related contexts. It may therefore be particularly useful when the objective is to monitor overall literacy levels, compare groups, or assess global changes following an educational intervention. However, the ECV value and PUC value indicate that the scale should not be interpreted as strictly unidimensional, supporting the continued relevance of domain-specific interpretation. Domain-specific scores provide a more precise picture of athletes’ literacy by distinguishing functional, interactive, and critical literacy (11, 12). Such scores are particularly useful for identifying specific educational needs and guiding targeted interventions (11, 14). ADL-S addresses a persistent gap in the field by providing a reliable and psychometrically validated measure to evaluate the effectiveness of prevention efforts (1, 15). Results of exploratory external correlates suggest that ADL-S scores may be associated with broader psychosocial processes involved in athletes’ responses to doping-risk situations. The negative associations with moral disengagement may suggest that some aspects of anti-doping literacy, such as appraising consequences and questioning justifications for prohibited substance use (11), could be inversely related to mechanisms that neutralise moral responsibility (30). Similarly, the positive association with resistive self-regulatory efficacy may reflect a possible link between anti-doping literacy and the perceived ability to apply knowledge under pressure (11, 29, 32, 33). The stronger associations observed for interactive literacy might be consistent with its more applied role in pressure and decision-making contexts (11). However, these exploratory associations should not be interpreted as evidence that moral disengagement or resistive self-regulatory efficacy are components or direct indicators of anti-doping literacy, nor as direct evidence of construct validity, and they require further confirmation.

Despite these strengths, this study has certain limitations. Although the sample size was sufficient for the analyses performed (17, 18), it was insufficient for more complex analyses, including the assessment of measurement invariance across gender, age, sport type, or competitive level. In addition, multi-source recruitment strategy, based partly on convenience, may limit generalization and, particularly to elite athletes or higher doping-risk context, as athletic status and WADA-related obligations were self-reported and could not be independently verified, especially among Prolific participants. As with all self-report measures, response biases must be considered. In anti-doping context, responses may be influenced by social desirability bias (42), and scores may partly reflect perceived compliance with anti-doping norms rather than actual competence. No social desirability control measure was included, and differences in the interpretation of items cannot be excluded despite expert analyses of the relevance and clarity of the items (43). In addition, exploratory external correlate analyses have certain limitations. The measures used were sport-specific rather than doping-specific, limiting their conceptual proximity to anti-doping literacy. Moreover, the correlational design precludes causal or directional conclusions, while limited prior evidence prevented strong *a priori* hypotheses. Finally, the modest associations observed, particularly for moral disengagement, warrant cautious interpretation.

The final measurement model included correlated residuals between some items. Although these correlations were theoretically interpretable, they may reflect local dependence or partial item redundancy. Future studies should examine whether they replicate in independent samples and whether item refinement is needed. Thus, temporal stability should be interpreted cautiously, as test-retest estimates were relatively low before outlier exclusion. Replication in larger and more diverse samples, using predefined procedures for handling influential observations, is needed. Although grounded in international anti-doping principles, its development with French-speaking athletes may limit its cross-cultural applicability, a cultural adaptation and validation are therefore needed before international use (44).

While these limitations restrict the current scope of the tool, they also open avenues for further research. Future studies should examine measurement invariance across gender, sport type and competitive level, and conduct cross-cultural and linguistic adaptations before ADL-S can be used for international comparisons or multinational programmes. Because the scale is based on self-report, future research should also include social desirability measures and complement ADL-S scores with more objective or scenario-based indicators of anti-doping competence. Future studies should examine these relationships using doping-specific measures, more robust theory-based hypotheses, and longitudinal designs to further support the construct validity of the ADL-S, clarify the direction of associations, and better characterize the nomological network surrounding anti-doping literacy. Finally, the incorporation of more dynamic framework, such as Sørensen et al. (10), could further capture contextual and systemic aspects of literacy.

ADL-S may offer new opportunities for both research and practice in the anti-doping context. At this stage, its potential applications should be considered in light of the present study's scope, which focused on the initial development and psychometric validation of the scale rather than on behavioural or longitudinal outcomes. Researchers could use ADL-S to assess changes in athletes’ anti-doping literacy and to examine whether different programme formats are associated with changes across the functional, interactive and critical literacy dimensions (14). ADL-S may enable educators, coaches, and sport organisations to generate detailed profiles of athletes’ literacy skills, guiding targeted interventions. At the policy level, the scale could contribute to monitoring and evaluation practices by national anti-doping organisations and federations in line with WADA's International Standard for Education, provided that its sensitivity to change and predictive validity are established in future research (12, 14). With cross-cultural adaptation and validation, ADL-S could make possible international comparison of anti-doping education, fostering a global evidence base for effective prevention strategies.

## Conclusion

The present study provides a solid foundation for the assessment of anti-doping literacy in sport. The ADL-S questionnaire developed is a psychometrically robust instrument that captures the functional, interactive, and critical dimensions of literacy, while also providing a global score. Based on the Doping Awareness Literacy (DAL) framework and health literacy models, it advances current knowledge by operationalising educational competencies in the anti-doping context. Beyond its psychometric strengths, ADL-S offers researchers and practitioners a reliable tool for assessing anti-doping literacy levels and the effectiveness of prevention and education programmes. It represents a meaningful step toward evidence-based approaches to doping prevention and the promotion of ethical decision-making in sport.

## Data Availability

The datasets presented in this study can be found in online repositories. The names of the repository/repositories and accession number(s) can be found below: https://osf.io/wpbmn.

## References

[1] World Anti-Doping Agency. International Standard for Education. Montreal: World Anti-Doping Agency (2021). Available online at: https://www.wada-ama.org/en/resources/world-anti-doping-code-and-international-standards/international-standard-education-ise (Accessed August 17, 2026).

[2] Mazanov J Backhouse S Connor J Hemphill D Quirk F. Athlete support personnel and anti-doping: knowledge, attitudes, and ethical stance. Scand J Med Sci Sport. (2014) 24(5):846–56. 10.1111/sms.12084 23692367

[3] Backhouse S. Anti-doping education for athletes. In: Møller V Waddington I Hoberman J, editors. Routledge Handbook of Drugs and Sport. London: Routledge (2015). p. 229–38. Available online at: https://www.taylorfrancis.com/chapters/edit/10.4324/9780203795347-24/anti-doping-education-athletes-susan-backhouse (Accessed August 17, 2026).

[4] UNESCO. International Convention Against Doping in Sport. Paris: UNESCO (2007). Available online at: https://www.unesco.org/en/legal-affairs/international-convention-against-doping-sport (Accessed August 17, 2026).

[5] Donovan RJ Egger G Kapernick V Mendoza J. A conceptual framework for achieving performance enhancing drug compliance in sport. Sport Med. (2002) 32(4):269–84. 10.2165/00007256-200232040-00005 11929355

[6] Williams TL Patterson LB Heyes AR Staff HR Boardley ID Petróczi A. Barriers and enablers in doping, anti-doping, and clean sport: a qualitative meta-synthesis informed by the theoretical domains framework and COM-B model. Psychol Sport Exerc. (2024) 72:102608. 10.1016/j.psychsport.2024.102608 38360079

[7] Backhouse SH Griffiths C McKenna J. Tackling doping in sport: a call to take action on the dopogenic environment. Br J Sports Med. (2018) 52(23):1485–6. 10.1136/bjsports-2016-097169 28666982

[8] Henriksen K Stambulova N Roessler KK. Holistic approach to athletic talent development environments: a successful sailing milieu. Psychol Sport Exerc. (2010) 11(3):212–22. 10.1016/j.psychsport.2009.10.005

[9] Filleul V d'Arripe-Longueville F Pavot D Bimes H Maillot J Meinadier E. Doping in elite cycling: a qualitative study of the underlying situations of vulnerability. Front Sport Act Living. (2024) 6:1482103. 10.3389/fspor.2024.1482103 PMC11620872 39650252

[10] Sørensen K Van den Broucke S Fullam J Doyle G Pelikan J Slonska Z. Health literacy and public health: a systematic review and integration of definitions and models. BMC Public Health. (2012) 12(1):1–13. 10.1186/1471-2458-12-80 PMC3292515 22276600

[11] Petroczi A Hudson A Allen M. Know your body. Know your substances. Know your rules: developing doping awareness literacy with the Safe You programme (2021). Available online at: https://osf.io/z72w3/ (Accessed August 17, 2026).

[12] Blank C Gatterer K Overbye M Schobersberger W Streicher B Petróczi A. GRADE IT—a literacy-based assessment tool for generating research-based assessment data to evidence the Impact of anti-doping education via athletes’ capability to make the right decision. Front Sport Act Living. (2022) 4:842192. 10.3389/fspor.2022.842192 PMC8965155 35368413

[13] Nutbeam D. Health literacy as a public health goal: a challenge for contemporary health education and communication strategies into the 21st century. Health Promot Int. (2000) 15(3):259–67. 10.1093/heapro/15.3.259

[14] Blank C Petróczi A. From learning to leading: making and evaluating the impact of anti-doping education with a competency approach. Soc Impacts. (2023) 1(1–2):100010. 10.1016/j.socimp.2023.100010

[15] Ntoumanis N Barkoukis V Pensgaard AM Ivarsson A Rivold JT Boardley ID. Development of brief assessment packages of psychosocial constructs related to doping. Drugs Educ Prev Policy. (2025) 32(6):575–83. 10.1080/09687637.2024.2423753

[16] Petróczi A Boardley ID. The meaning of “clean” in anti-doping education and decision making: moving toward integrity and conceptual clarity. Front Sport Act Living. (2022) 4:869704. 10.3389/fspor.2022.869704 PMC9136326 35647537

[17] Boateng GO Neilands TB Frongillo EA Melgar-Quiñonez HR Young SL. Best practices for developing and validating scales for health, social, and behavioral research: a primer. Front Public Heal. (2018) 6:149. 10.3389/fpubh.2018.00149 PMC6004510 29942800

[18] Stefana A Damiani S Granziol U Provenzani U Solmi M Youngstrom EA. Psychological, psychiatric, and behavioral sciences measurement scales: best practice guidelines for their development and validation. Front Psychol. (2025) 15:1494261. 10.3389/fpsyg.2024.1494261 39916786 PMC11798685

[19] Simms LJ Zelazny K Williams TF Bernstein L. Does the number of response options matter? Psychometric perspectives using personality questionnaire data. Psychol Assess. (2019) 31(4):557–67. 10.1037/pas0000648 30869956

[20] Vallerand RJ. Vers une méthodologie de validation trans-culturelle de questionnaires psychologiques: implications pour la recherche en langue française. Can Psychol. (1989) 30(4):662–80. 10.1037/h0079856 6850979

[21] Mirzaei A Carter SR Patanwala AE Schneider CR. Missing data in surveys: key concepts, approaches, and applications. Res Soc Adm Pharm. (2022) 18(2):2308–16. 10.1016/j.sapharm.2021.03.009 33775556

[22] Myers ND Martin JJ Ntoumanis N Celimli S Bartholomew KJ. Exploratory bifactor analysis in sport, exercise, and performance psychology: a substantive-methodological synergy. Sport Exerc Perform Psychol. (2014) 3(4):258–72. 10.1037/spy0000015

[23] Alavi M Visentin DC Thapa DK Hunt GE Watson R Cleary M. Chi-square for model fit in confirmatory factor analysis. J Adv Nurs. (2020) 76(9):2209–11. 10.1111/jan.14399 32323338

[24] Béland S Cousineau D Loye N. Utiliser le coefficient omega de McDonald à la place de l’alpha de Cronbach. McGill J Educ. (2017) 52(3):791–804. 10.7202/1050915ar

[25] Bollen KA. When good loadings go bad: robustness in factor analysis. Struct Equ Modeling. (2020) 27(4):515–30. 10.1080/10705511.2019.1691005 36381611 PMC9645687

[26] Rodriguez A Reise S Haviland M. Evaluating bifactor models: calculating and interpreting statistical indices. Psychol Methods. (2016) 21(2):137–53. 10.1037/met0000045 26523435

[27] Reise SP. The rediscovery of bifactor measurement models. Multivariate Behav Res. (2012) 47(5):667–96. 10.1080/00273171.2012.715555 24049214 PMC3773879

[28] Boardley ID Grix J Dewar AJ. Moral disengagement and associated processes in performance-enhancing drug use: a national qualitative investigation. J Sports Sci. (2014) 32(9):836–44. 10.1080/02640414.2013.862842 24405120

[29] Ring C Kavussanu M. The role of self-regulatory efficacy, moral disengagement and guilt on doping likelihood: a social cognitive theory perspective. J Sports Sci. (2018) 36(5):578–84. 10.1080/02640414.2017.1324206 28481691

[30] Bandura A Barbaranelli C Caprara GV Pastorelli C. Mechanisms of moral disengagement in the exercise of moral agency. J Pers Soc Psychol. (1996) 71(2):364–74. 10.1037/0022-3514.71.2.364

[31] Corrion K Long T Smith AL d’Arripe-Longueville F. It’s not my fault; it’s not serious”: athlete accounts of moral disengagement in competitive sport. Sport Psychol. (2009) 23(3):388–404. 10.1123/tsp.23.3.388

[32] Boardley ID Smith AL Mills JP Grix J Wynne C. Empathic and self-regulatory processes governing doping behavior. Front Psychol. (2017) 8(SEP):1–11. 10.3389/fpsyg.2017.01495 29018370 PMC5614971

[33] Corrion K Scoffier Meriaux S Longueville F. Self-regulatory mechanisms of doping intentions in elite athletes: the role of self-determined motivation in sport. J Sports Med Doping Stud. (2017) 7:4. 10.4172/2161-0673.1000197

[34] Corrion K Scoffier S Gernigon C Cury F D’Arripe-Longueville F. Development and factorial validity of a moral disengagement in sport short scale. Encephale. (2010) 36(6):495–503. 10.1016/j.encep.2010.03.003 21130234

[35] Corrion K Gernigon C Debois N D’Arripe-Longueville F. Factor validity and reliability of the resistive self-regulatory efficacy in sport scale (rsress) in a French sample. Int J Sport Psychol. (2013) 44(2):128–44. 10.7352/IJSP.2013.44.128

[36] Schober P Boer C Schwarte LA. Correlation coefficients: appropriate use and interpretation. Anesth Analg. (2018) 126(5):1763–8. 10.1213/ANE.0000000000002864 29481436

[37] Koo TK Li MY. A guideline of selecting and reporting intraclass correlation coefficients for reliability research. J Chiropr Med. (2016) 15(2):155–63. 10.1016/j.jcm.2016.02.012 27330520 PMC4913118

[38] Park MS Kang KJ Jang SJ Lee JY Chang SJ. Evaluating test-retest reliability in patient-reported outcome measures for older people: a systematic review. Int J Nurs Stud. (2018) 79:58–69. 10.1016/j.ijnurstu.2017.11.003 29178977

[39] Bland JM Altman DG. Statistical methods for assessing agreement between two methods of clinical measurement. Lancet. (1986) 327(8476):307–10. 10.1016/S0140-6736(86)90837-8 2868172

[40] Tukey JW. Exploratory data analysis: past, present and future. In: Proceedings of the Special Session on Information Theory and Data Analysis. Princeton, NJ: Princeton University (1997). p. 131–60. 10.1007/978-3-031-20719-8_2

[41] Vinutha HP Poornima B Sagar BM. Detection of outliers using interquartile range technique from intrusion dataset. In: Information and Decision Sciences: Proceedings of the 6th International Conference on ficta. Singapore: Springer (2018). p. 511–8. 10.1007/978-981-10-7563-6_53

[42] Bernardi RA Nash J. The importance and efficacy of controlling for social desirability response bias. Ethics Behav. (2023) 33(5):413–29. 10.1080/10508422.2022.2093201

[43] Davidov E Meuleman B Cieciuch J Schmidt P Billiet J. Measurement equivalence in cross-national research. Annu Rev Sociol. (2014) 40(1):55–75. 10.1146/annurev-soc-071913-043137

[44] Cruchinho P López-Franco MD Capelas ML Almeida S Bennett PM Miranda da Silva M. Translation, cross-cultural adaptation, and validation of measurement instruments: a practical guideline for novice researchers. J Multidiscip Healthc. (2024) 17:2701–28. 10.2147/JMDH.S419714 38840704 PMC11151507

