# Supplementary material for: Anti-doping literacy scale (ADL-S): development and validation of a French language instrument based on the doping awareness literacy framework
Source: Front Sports Act Living. 2026 Sep 15;8:1810415. doi: 10.3389/fspor.2026.1810415 (PMC13619928; doi:10.3389/fspor.2026.1810415)
Supplement: Supplementary file 1 [file Table1.docx]

Supplementary Table 1. French version and English translation of the Anti-Doping Literacy Scale (ADL-S) items.

| Dimensions | French version | English translation |
| --- | --- | --- |
| **Functional literacy** | | |
| LF1 | Je comprends les principales Violations des Règles Anti-Dopage (VRAD). | I understand the main Anti-Doping Rule Violations. |
| LF2 | Je sais ce que représente ma responsabilité personnelle (objective) concernant les substances que je consomme ou qui peuvent être détectées dans mon corps. | I know what my personal (Strict Liability) responsibility is regarding the substances I consume or that may be detected in my body. |
| LF3 | Je comprends le cadre d’utilisation des Autorisations d’Usages à des fins Thérapeutiques (AUT). | I understand the framework for using Therapeutic Use Exemptions (TUEs). |
| LF4 | Je comprends l'intérêt du système d’administration et de gestion antidopage (ADAMS). | I understand the purpose of the Anti-Doping Administration and Management System (ADAMS). |
| LF5 | Je suis conscient.e des risques que l’utilisation de substances et/ou méthodes interdites peut avoir sur mes relations avec mes coéquipiers, coéquipières ou ami(e)s. | I am aware of the risks that using prohibited substances and/or methods may pose to my relationships with teammates or friends. |
| LF6 | Je suis conscient.e des conséquences possibles liées à l'utilisation de substances et/ou des méthodes interdites, telles que des sanctions ou interdiction d'entrainement en club. | I am aware of the possible consequences of using prohibited substances and/or methods, such as sanctions or being banned from training with my club. |
| LF7 | Je comprends les conséquences que l’utilisation de substances et/ou de méthodes interdites peuvent avoir sur mes résultats sportifs. | I understand the consequences that using prohibited substances and/or methods may have on my athletic performance. |
| LF8 | Je suis conscient.e des risques de développer une dépendance à certains produits dopants en cas d’utilisation de ces derniers. | I am aware of the risk of developing a dependence on certain doping products if I use them. |
| **Interactive literacy** | | |
| LI1 | Je me sens capable de faire face et/ou de récupérer d’une blessure importante sans être tenté(e) d’utiliser des substances et/ou des méthodes interdites. | I feel able to cope with and/or recover from a serious injury without being tempted to use prohibited substances and/or methods. |
| LI2 | Je me sens capable d’être performant(e) malgré des signes de fatigue sans être tenté(e) d’utiliser des substances et/ou des méthodes interdites. | I feel able to perform well despite feeling tired, without being tempted to use prohibited substances and/or methods. |
| LI3* | Je me sens incapable de continuer à m’entraîner en cas de fatigue sans être tenté(e) d’utiliser des substances et/ou des méthodes interdites. | I feel unable to keep training when I’m tired without being tempted to use prohibited substances and/or methods. |
| LI4 | Je me sens capable de faire face à des maladies courantes, sans être tenté(e) d’utiliser des substances et/ou des méthodes interdites. | I feel able to deal with common illnesses without being tempted to use prohibited substances and/or methods. |
| LI5 | Je me sens capable de faire face à une charge importante d’entrainement, sans être tenté(e) d’utiliser des substances et/ou des méthodes interdites. | I feel able to handle a heavy training load without being tempted to use prohibited substances and/or methods. |
| LI6 | Je me sens capable de résister à la pression de mon entourage familial sans être tenté(e) d’utiliser des substances et/ou des méthodes interdites | I feel able to resist pressure from my family environment without being tempted to use prohibited substances and/or methods. |
| LI7 | Je me sens capable de faire face à des échecs sportifs ou des baisses de performance, sans être tenté(e) d’utiliser des substances et/ou des méthodes interdites | I feel able to cope with sporting failures or performance drops without being tempted to use prohibited substances and/or methods. |
| LI8 | Je me sens capable de résister à la pression de mon entourage sportif sans être tenté(e) d’utiliser des substances et/ou des méthodes interdites | I feel able to resist pressure from my sporting environment without being tempted to use prohibited substances and/or methods. |
| LI9* | Je me sens incapable de rester lucide lorsque je suis fatigué(e) physiquement et/ou mentalement, sans être tenté(e) d’utiliser des substances ou méthodes interdites | I feel unable to stay clear-headed when physically and/or mentally fatigued, without being tempted to use prohibited substances and/or methods. |
| LI10 | Je me sens capable de faire face à un épuisement physique et/ou mental, sans être tenté(e) d’utiliser des substances ou méthodes interdites | I feel able to deal with physical and/or mental exhaustion without being tempted to use prohibited substances and/or methods. |
| **Critical literacy** | | |
| LC1 | J'ai confiance en ma capacité à expliquer les enjeux de l’antidopage à d’autres athlètes | I am confident in my ability to explain anti-doping issues to other athletes. |
| LC2 | J'ai confiance en ma capacité à utiliser la plateforme de signalement pour déclarer un(e) athlète dopé(e). | I am confident in my ability to use the reporting platform to report a doped athlete. |
| LC3 | J'ai confiance en ma capacité à discuter de la prévention du dopage avec d’autres athlètes | I am confident in my ability to talk about doping prevention with other athletes. |
| LC4 | J'ai confiance en ma capacité à expliquer les règles antidopage à d'autres athlètes | I am confident in my ability to explain anti-doping rules to other athletes. |
| LC5 | Je suis confiant(e) dans le fait que mes actions puissent contribuer à promouvoir un sport propre | I am confident that my actions can help promote clean sport. |

Notes. LF = Functional literacy; LI = Interactive literacy; LC = Critical literacy. * These items were removed and are not part of the final model.
